# Veterinary blood culture diagnostic testing: methodology and results from a German single-center study on dogs (2014-2022)

**DOI:** 10.1093/jvimsj/aalaf057

**Published:** 2026-01-21

**Authors:** Teresa Sandbrink, Antina Lübke-Becker, Caroline Robé, Marcus Fulde, Myriam Antonia Knopf, Roswitha Merle, Christiane Weingart, Uwe Rösler, Stefan Schwarz, Barbara Kohn

**Affiliations:** Small Animal Clinic, Freie Universität Berlin, Berlin 14163, Germany; Veterinary Centre for Resistance Research, Freie Universität Berlin, Berlin 14163, Germany; Veterinary Centre for Resistance Research, Freie Universität Berlin, Berlin 14163, Germany; Institute of Microbiology and Epizootics, Freie Universität Berlin, Berlin 14163, Germany; Veterinary Centre for Resistance Research, Freie Universität Berlin, Berlin 14163, Germany; Institute for Animal Hygiene and Environmental Health, Freie Universität Berlin, Berlin 14163, Germany; Veterinary Centre for Resistance Research, Freie Universität Berlin, Berlin 14163, Germany; Institute of Microbiology and Epizootics, Freie Universität Berlin, Berlin 14163, Germany; Small Animal Clinic, Freie Universität Berlin, Berlin 14163, Germany; Veterinary Centre for Resistance Research, Freie Universität Berlin, Berlin 14163, Germany; Institute of Veterinary Epidemiology and Biostatistics, Freie Universität Berlin, Berlin 14163, Germany; Small Animal Clinic, Freie Universität Berlin, Berlin 14163, Germany; Veterinary Centre for Resistance Research, Freie Universität Berlin, Berlin 14163, Germany; Institute for Animal Hygiene and Environmental Health, Freie Universität Berlin, Berlin 14163, Germany; Veterinary Centre for Resistance Research, Freie Universität Berlin, Berlin 14163, Germany; Institute of Microbiology and Epizootics, Freie Universität Berlin, Berlin 14163, Germany; Small Animal Clinic, Freie Universität Berlin, Berlin 14163, Germany; Veterinary Centre for Resistance Research, Freie Universität Berlin, Berlin 14163, Germany

**Keywords:** antimicrobial resistance, bacteria, microbiological diagnostics, septicemia, susceptibility

## Abstract

**Background:**

Blood cultures (BCs) are the gold standard for identifying bacterial bloodstream infections and are vital for directing antimicrobial treatment.

**Hypothesis/Objectives:**

Characterize BC results and diagnostic testing in veterinary medicine. Pathogen identification (ID) results, antimicrobial susceptibility testing (AST) results, and management factors were investigated to identify optimization opportunities.

**Animals:**

A total of 750 BCs from 687 dogs presented between January 2014 and September 2022 at a German teaching hospital, with 102 positive BCs from 101 dogs.

**Methods:**

Retrospective analysis of historical data. A BC system suitable for aerobic and anaerobic pathogens was used.

**Results:**

Blood culture yield (clinically relevant growth in 102/750 BCs) was 13.6%, and the contamination rate was 1.9%. Commonly isolated pathogens were Enterobacterales (*n* = 15/25 multidrug-resistant), coagulase-positive staphylococci (*n* = 3/23 methicillin-resistant), beta-hemolytic streptococci (*n* = 19), and obligate anaerobes (*n* = 22). Polymicrobial growth occurred in 8 BCs (7.8%). Median duration of the preanalytical phase (sampling until receipt in laboratory) was 1 day (interquartile range [IQR], 1-2), from receipt in laboratory until ID results 1 day (IQR, 1-2), and until AST results 2 days (IQR, 2-4). The preanalytical phase of BCs taken on weekends was a median of 1 day longer *(P* < .001). Dogs treated with antimicrobials within 7 days before sampling were 10.74 times more likely to yield a multidrug-resistant isolate (95% confidence interval [CI], 3.62-31.86).

**Conclusions and clinical importance:**

Improving BC processing requires addressing the management factors BC yield, contamination rate, weekend sampling, and antimicrobial pretreatment. Detection of resistant bacteria emphasizes the need for more rapid processing to shorten the time to optimized antimicrobial treatment.

## Introduction

Blood cultures (BCs) are the gold standard for identifying bacterial bloodstream infections (BSIs), a critical condition associated with a high mortality rate^1^ in humans and animals.^2^^,^^3^ Consisting of pathogen identification (ID) and antimicrobial susceptibility testing (AST), BC diagnostic tests form the basis for optimized antimicrobial treatment.^4^^,^^5^ Rapid and correct diagnosis of BSIs in the form of sepsis is crucial for clinicians to make informed decisions and for the patient’s prognosis.^6^^,^^7^ Management factors such as the contamination rate have a clinically relevant impact on the quality of BCs. Blood culture contamination has a negative impact on patient care^8^ by extending hospital stays,^9^ increasing treatment costs, and possibly leading to unnecessary procedures^10^^,^^11^ and the inappropriate use of antibiotics, putting patients at higher risk for iatrogenic infections.^11^ To optimize the management of patients with BSIs, all clinical aspects should be considered. Insight on the infection source and causative pathogen could be provided by microbiological culture results from other sampling sites performed during the same presentation episode.^12–14^

However, few studies pertaining to BCs have been conducted in veterinary medicine, with no current European data being available. The few available studies mainly focused on determining the prevalence of isolated pathogens and their characteristics, including antimicrobial resistance profiles.^3^^,^^4^^,^^15^ Possible influences on the BC yield have been investigated,^12^^,^^16^ but their impact on sample processing duration has not been analyzed.

In summary, information on BC results and management in veterinary medicine is limited. Therefore, our retrospective study aimed to analyze BC results and elucidate their processing in veterinary medicine. We hypothesized that prior antimicrobial treatment, sampling on weekends, polymicrobial growth, and Gram-positive growth might result in longer sample processing duration. It was investigated whether prior antimicrobial treatment led to a higher yield of multidrug resistant (MDR) pathogens. Culture results from other sampling sites were compared to BC results to better understand their role in guiding antimicrobial treatment.

## Materials and methods

### Inclusion criteria and data collection

All BCs from dogs processed from January 2014, when the use of matrix-assisted laser desorption/ionization time-of-flight mass spectrometry (MALDI-ToF MS; Bruker Daltonics, Billerica, USA) was established for ID in routine diagnostic testing, until September 2022 were considered for our retrospective study, and positive BCs were included. Analyzed data included background information (if available, Table S1) of dogs, BC results, culture results from other sampling sites during the same presentation episode (if available), and dates of (1) sample collection, (2) receipt in the clinical microbiology laboratory, and (3) reporting of ID and AST results. Data were obtained from the data storage system of the clinical microbiology laboratory of the Institute of Microbiology and Epizootics and digital patient records of the Small Animal Clinic of the Freie Universität Berlin, a teaching hospital treating both primary care and referral patients. To ensure that only true-positive cultures were included in the analysis, possible contaminations were identified as follows: Bacteria commonly found in the microbiota or environment (*Bacillus* spp., coagulase-negative staphylococci, *Streptococcus mitis* group, *Moraxella* spp., *Leuconostoc lactis*) were classified as contaminants if the clinical context did not indicate the isolate caused sepsis, they were not found in other cultures from the same presentation episode, and the patient was not considered at high risk (eg, immunocompromised patients). Isolates of bacterial species more frequently reported as causative pathogens (*Acinetobacter ursingii, Pasteurella canis*, and *Clostridium haemolyticum*) and those more commonly associated with infections (*Staphylococcus intermedius* group) were considered contaminants if there was limited growth (in terms of quantity and time to positivity), they were not isolated in other cultures from the same presentation episode, and the clinical context did not indicate their involvement in sepsis.^2^^,^^17^^,^^18^

### Sample collection and processing

A manual BC system (Signal Blood Culture System, Oxoid, Waltham, USA), in which bottles are inspected for visual signs of growth,^19^ was used. A single BC bottle was inoculated with a blood sample collected aseptically from each patient. Blood cultures were preincubated at 37°C in-clinic before transport to the clinical microbiology laboratory several times a day during working hours (walking distance 5 min). Upon receipt in the laboratory, the date of arrival was documented. Working hours of the laboratory were from 9:00 am until 4:00 pm during weekdays and until 12:00 pm on Saturdays. Blood culture samples were only transported to the laboratory during these times and placed into laboratory incubators as soon as possible. In order to investigate the diagnostic process of BCs, sample processing was divided into a preanalytical and an analytical phase. The preanalytical phase was defined as the time from the date of sampling until receipt of the sample in the laboratory. The analytical phase was defined as the time from the date of sample receipt until complete reporting of ID and AST results. Samples were incubated at 37°C for ≥ 18 h from time of sampling or until a positive BC signal was observed. Overnight subculture was performed as described previously,^20^ with the addition of chocolate agar (Thermo Scientific, Wesel, Germany) for the cultivation of fastidious bacteria for 24-48 h (37°C, microaerobic, 7% CO_2_). Pathogen ID and AST were carried out from pure bacterial colonies. Final subculture of negatively signaling BC bottles was performed after 5 days. Pathogen ID was performed using MALDI-ToF MS. Antimicrobial susceptibility testing was performed by broth microdilution according to the Clinical and Laboratory Standards Institute (CLSI) standards valid at the time of sample processing using Micronaut-S Kleintier plates (Bruker Daltonics, Billerica, USA) or the VITEK 2 system (bioMérieux, Marcy-l’Étoile, France), depending on the bacterial pathogen. Antimicrobial susceptibility testing results were reported using the categories: “S”—susceptible, “I”—intermediate (if available), or “R”—resistant. Because no CLSI-approved breakpoints are available for blood samples from dogs, dog-specific breakpoints applicable to other infection sites (preferably skin and soft tissue infections) were applied. When no dog-specific breakpoints were available, CLSI breakpoints applicable to other animal species or humans were used. No routine AST was performed on obligate anaerobic isolates because of their probable susceptibility to commonly used antimicrobial agents. For our study, isolates were considered MDR when phenotypic testing showed that they were not susceptible to antimicrobial agents of ≥ 3 different classes,^21^ excluding intrinsic resistances. For *Klebsiella pneumoniae, Escherichia coli*, and *Proteus mirabilis,* testing for extended-spectrum β-lactamase (ESBL) production and for staphylococci, testing for methicillin resistance with oxacillin (*Staphylococcus pseudintermedius, Staphylococcus coagulans*) or cefoxitin (*Staphylococcus aureus*) was performed according to CLSI standards.^22^^,^^23^ To add perspective to the report of antimicrobial resistances in our study, the categories “A”—“avoid” (which must not be used in animals, according to European Union regulation 2022/1255;^24^ reported in the study: carbapenems), “B”—“restrict” (reported in the study: third-generation cephalosporins and fluoroquinolones), “C”—“caution” (reported in the study: aminoglycosides except spectinomycin, aminopenicillins in combination with β-lactamase inhibitors, first- and-second generation cephalosporins, macrolides, lincosamides, and phenicols), and “D”—“prudence” (reported in our study: aminopenicillins, tetracyclines, and sulfonamides with or without trimethoprim) of antimicrobial agents created by the European Medicines Agency (EMA) were used (see Table S1).^25^ Positive and negative BC results were saved in the data storage system of the clinical microbiology laboratory and uploaded into the digital patient records of the clinic. Cultures from other sampling sites were performed correspondingly, as described above. Concordance of pathogens from BCs and cultures from other sampling sites was defined as identical ID and AST results.

### Statistical analysis

Descriptive statistics were performed using IBM SPSS Statistics (Version 27; Armonk, New York, USA). All available data in the study period were included in terms of a convenience sample. The data distributions for continuous variables (including duration of sample processing steps) were evaluated for normality by visual inspection. Because duration data were not normally distributed, statistical analyses were performed using the Mann–Whitney U test to compare the duration of specific processing steps between groups. To examine the association of antimicrobial pretreatment and the isolation of MDR bacteria, the chi-squared test was applied and the odds ratio including 95% CI was calculated. A *P*-value < .05 was considered statistically significant. To calculate the negative predictive value (NPV) of the BC results, in the absence of a definitive gold standard, we used the final reported result for each BC as a “pseudo-gold standard” (Table S2). Throughout this analysis, the NPV refers to the comparison of the initial result to the subsequent results. The NPV of the BC results was calculated by dividing the number of “true negatives” (BCs that were initially reported negative without later amendment) by the added number of “true negatives” and “false negatives” (BCs that were initially reported negative but were subsequently reported positive) and a CI was calculated (CI Calculator: Diagnostic Statistics, The Chinese University of Hong Kong, Hong Kong).

## Results

### Numbers of BCs included in the study

Of the 750 BCs from dogs submitted by the clinic for processing in the clinical microbiology laboratory, 116 were positive for ≥ 1 pathogen. Blood cultures were determined to be false-positive because of contamination in 14 cases; these BCs were not included in the analysis. The contamination rate was 1.9%. Thus, 102 BCs from 101 dogs (2 BCs were taken from 1 dog) were included in the analyses. The BC yield (102 BCs with clinically relevant growth from 750 BCs) was 13.6%.

### Patient data

The mean age of the 101 patients was 8 years (IQR 5.2-11.5). These included 21 (20.8%) neutered males, 31 (30.7%) intact males, 30 (29.7%) spayed females, and 19 (18.8%) intact females. In 30 cases, the dog was mixed breed (29.7%), in 71 cases purebred (70.3%). The most common breeds were Golden Retriever and Bernese Mountain Dog (*n* = 4, each), followed by Labrador Retriever, Jack Russell Terrier, German Shepherd, Rhodesian Ridgeback, Great Dane, Dachshund, and Shih Tzu (*n* = 3, each [Table S1]). Antimicrobials had been administered to 41 (40.2%) of the dogs in the 7 days before sampling. Most commonly administered antimicrobial agents were a combination of amoxicillin and clavulanic acid (*n* = 16), followed by doxycycline (*n* = 7 [Table S1]). For 3 dogs, the antimicrobial agent administered was unknown. For 2 dogs, sufficient data were not available. Of the 59 (57.8%) dogs that did not receive antimicrobials in the 7 days before sampling, 6 had a history of antimicrobial administration within 30 days before sampling (Table S1).

### Blood culture results

In 83 of the 102 BCs (81.4%), the first ID result was reported on day 1 after the day of sampling. In the remaining BCs, the first result was reported on day 2 (*n* = 5), day 3 (*n* = 12), or day 4 (*n* = 2) after sampling. Initially, 17 of the 102 positive BCs were reported as negative (16 on day 1 and 1 on day 3 of the analytical phase). In these 17 initially negative BC cases, ID results were reported up to 6 days after the receipt of the sample in the laboratory (Table 1). In 97.4% of the cases, the negative result was not amended (NPV, 0.974; 95% CI, 0.96-0.98).

**Table 1:** Day of complete pathogen identification (ID) results from the day of sampling (day 0) for monomicrobial and polymicrobial blood cultures from dogs with clinically relevant pathogens (*n* = 102).

**Results**	**Days until of complete ID results (from day 0 of sampling)**						
	Day 1	Day 2	Day 3	Day 4	Day 5	Day 6	Total
**Initial result: Negative**							
**Final result: Monomicrobial**		7	5	1	1	3	17
**Initial result: Monomicrobial**							
** Final result: Monomicrobial**	61	6	8	2			77
** Final result: Polymicrobial (2 species)**	1		1	1	1		4
**Initial result: Polymicrobial (2 species)**							
** Final result: 2 species**		1		2			3
** Final result: 3 species**		1					1

Polymicrobial growth occurred in 8 BCs. In 4 of the 81 BCs that initially were reported as monomicrobial, a second pathogen was reported (day 2 [*n* = 1]; day 3 [*n* = 1]; day 4 [*n* = 1]; and day 5 [*n* = 1]). Polymicrobial growth with 2 clinically relevant pathogens was initially reported in 4 BCs. In 1 BC, an additional pathogen was reported, for a total of 3 pathogens.

### Sample processing

**Figure 1:**
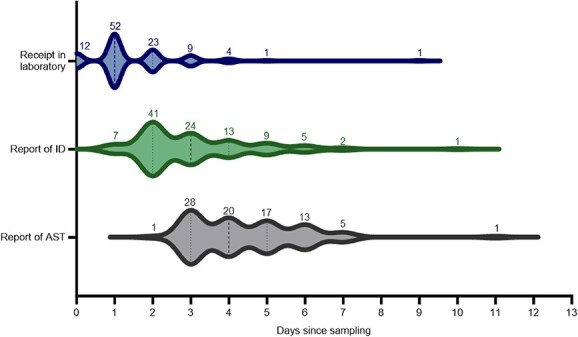
Time (in days) of individual sample processing steps and feedback of results of clinically relevant positive BCs (*n* = 102) from the day of sample collection, including reception in clinical microbiology laboratory, report of pathogen ID results, and report of AST results. Plots are amended with numbers indicating the number of samples on the respective day: median (dashed line); first and third quartiles (dotted line). Abbreviations: AST = antimicrobial susceptibility testing; BCs = blood cultures; ID = identification.

The median duration from the day of sampling until complete result reporting was 4 days (IQR 3-5). The median duration of the preanalytical phase was 1 day (IQR, 1-2), that of the analytical phase was 2 days (IQR, 2-4). The analytical phase consisted of the time until final ID results in all, with AST results provided in 85 cases. The median duration until final ID result reporting was 1 day (IQR, 1-2) and until AST result reporting 2 days (IQR, 2-4) from receipt in the laboratory (Figure 1).

Multiple factors influenced sample processing duration. Sampling on weekends (Friday, Saturday, or Sunday) significantly affected the processing steps: the preanalytical phase was a median of one day longer (*P* < .001), the time until ID and AST shorter (by a median of 0 [*P* = .02] and 2 days [*P* < .001]). However, total sample processing duration did not differ significantly from samples taken on weekdays (median of 1 day shorter; *P* = .34). The duration until ID was a median of 2.5 days longer for polymicrobial cultures (*P* = .001), but other steps were not significantly affected (preanalytical phase: 0 days shorter, *P* = .07; AST: 1 day longer, *P* = .42). Antimicrobial administration in the 7 days before sampling did not significantly influence the duration of any processing steps, with none of the median durations differing (preanalytical phase: *P* = .3; ID: *P* = .59; AST: *P* = .65). Samples in which Gram-positive pathogens were isolated did not have a significantly longer sample processing duration than samples in which Gram-negative pathogens were isolated, with none of the median durations differing (preanalytical phase: *P* = .08; time until ID: *P* = .31; AST: *P* = .91).

### Pathogen identification

In 69 of the 102 clinically relevant BCs, only Gram-positive bacteria were isolated (67.7%), in 29 (28.4%) only Gram-negative bacteria were isolated (Table 2), whereas Gram-positive and Gram-negative growth was found in 4 (3.9%) BCs. Monomicrobial growth was found in 94 (92.2%) and polymicrobial growth in 8 (7.9%) of the 102 BCs, with 2 pathogens being isolated in 7 BCs and 3 pathogens in 1 BC.

**Table 2:** Clinically relevant bacterial species from positive blood cultures (*n* = 102).

Species Isolated		Number (%)
**Monomicrobial**	**94 (91.26)**	
** Enterobacterales**	**22 (21.57)**	
*** Escherichia coli***		18 (17.65)
*** Klebsiella pneumoniae***		2 (1.96)
*** Enterobacter cloacae***		1 (0.98)
*** Salmonella enterica* spp. *enterica***		1 (0.98)
** Coagulase-positive *Staphylococcus* spp.**	**21 (20.59)**	
*** Staphylococcus aureus***		4 (3.92)
*** Staphylococcus coagulans***		1 (0.98)
*** Staphylococcus pseudintermedius***		16 (15.69)
** β-hemolytic *Streptococcus* spp.**	**17 (16.67)**	
*** Streptococcus canis***		15 (14.71)
*** Streptococcus equi* spp. *zooepidermicus***		2 (1.96)
** Obligate anaerobic species**	**16 (15.69)**	
*** Bacteroides fragilis***		1 (0.98)
*** Clostridium perfringens***		13 (12.75)
*** Clostridium* sp.**		1 (0.98)
*** Prevotella* sp.**		1 (0.98)
*** Enterococcus* spp.**	**8 (7.84)**	
***Enterococcus avium***		1 (0.98)
*** Enterococcus faecalis***		3 (2.94)
*** Enterococcus faecium***		3 (2.94)
*** Enterococcus hirae***		1 (0.98)
*** Pasteurella* spp.**	**5 (4.9)**	
*** Pasteurella canis***		3 (2.94)
*** Pasteurella multocida***		1 (0.98)
*** Pasteurella* sp.**		1 (0.98)
** α-hemolytic *Streptococcus* spp.**	**3 (2.94)**	
*** Streptococcus gallolyticus***		2 (1.96)
*** S. gallolyticus* ssp. *pasteurianus***		1 (0.98)
*** Ersipelothrix* spp.**	**2 (1.96)**	
*** Erysipelothrix rhusiopathiae***		2 (1.96)
**Polymicrobial**	**8 (7.84**)	
** Two species isolated**	**7 (6.86)**	
*** C. perfringens* + *Clostridium butyricum***		1 (0.98)
*** C. perfringens* + *E. coli***		1 (0.98)
*** C. perfringens* + *K. pneumoniae***		1 (0.98)
*** C. perfringens* + *Pseudomonas aeruginosa***		1 (0.98)
*** E. faecium* + *Proteus mirabilis***		1 (0.98)
*** E. faecium* + *S. aureus***		1 (0.98)
*** S. pseudintermedius* + *S. canis***		1 (0.98)
** Three species isolated**	**1 (0.98)**	
*** C. perfringens* + *S. canis* + *E. hirae***		1 (0.98)
		**102 (100)**

The most common bacterial isolates from monomicrobial BCs were Enterobacterales (*n* = 22), followed by coagulase-positive *Staphylococcus* spp. (*n* = 21), β-hemolytic *Streptococcus* spp. (*n* = 17), and obligate anaerobic species (*n* = 16). Furthermore, *Enterococcus* spp. (*n* = 8), *Pasteurella* spp. (*n* = 5), α-hemolytic *Streptococcus* spp. (*n* = 3), and *Erysipelothrix rhusiopathiae* (*n* = 2) were isolated. The most common species isolated in polymicrobial BCs was *Clostridium perfringens* (Table 2).

**Figure 2:**
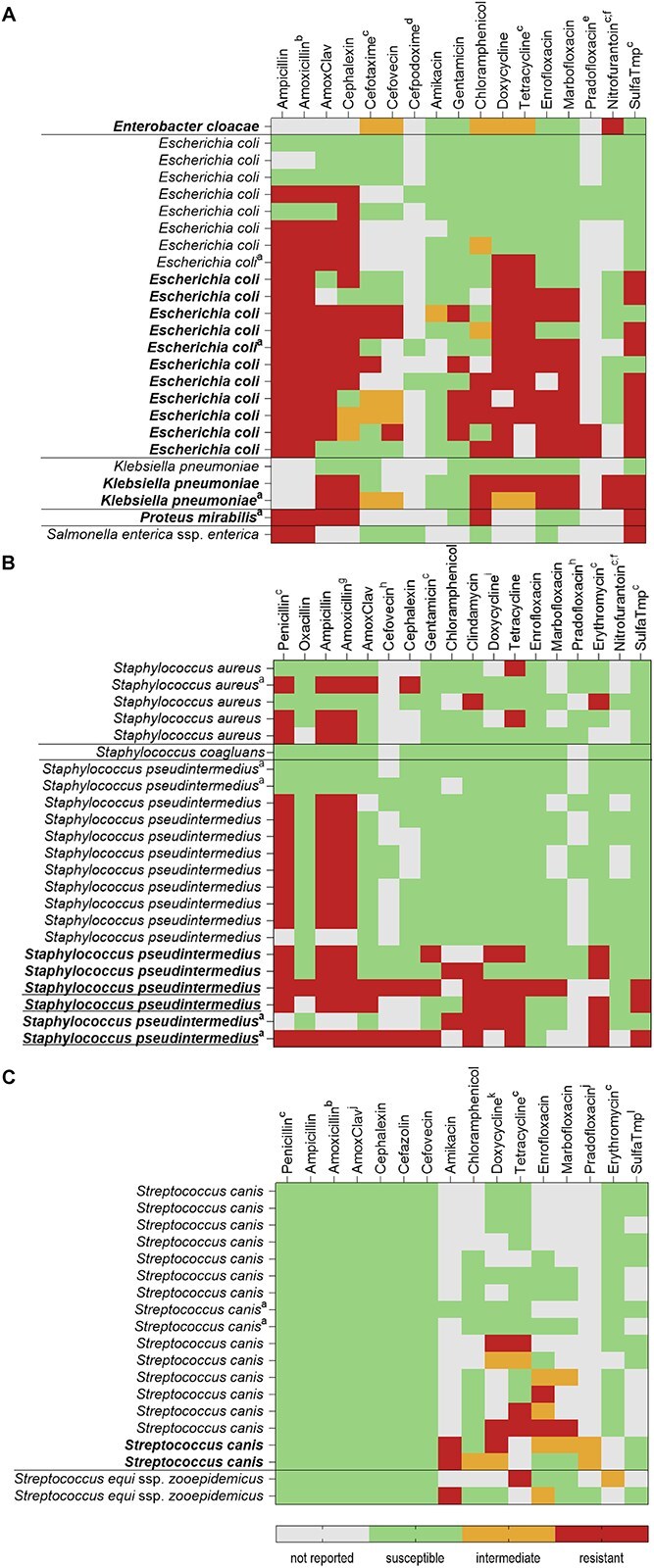
Antimicrobial susceptibility testing results of the 3 most commonly isolated groups from positive blood cultures from dogs in the years 2014-2022. (A) Enterobacterales; (B) coagulase-positive *Staphylococcus* spp.; (C) β-hemolytic *Streptococcus* spp. Testing performed to Clinical and Laboratory Standards Institute standards. Intrinsic resistances not depicted. Multidrug-resistant isolates indicated in bold, methicillin-resistant isolates indicated with underlining. ^a^isolated from polymicrobial blood cultures (not indicated = monomicrobial blood cultures), ^b^inferred from ampicillin, ^c^human breakpoint, ^d^breakpoint for *Escherichia coli* and *Proteus mirabilis*, ^e^breakpoint for *E. coli*, ^f^lower urine tract breakpoint, ^g^inferred from penicillin, ^h^breakpoint for *Staphylococcus pseudintermedius*, ^i^susceptibility inferred from tetracycline, ^j^cat breakpoint, ^k^breakpoint for *Streptococcus equi* subsp. *equi* and subsp. *zoopepidemicus* from horses, ^l^breakpoint for *Streptococcus pneumoniae*.

### Antimicrobial susceptibility testing

The AST results were reported for 89 isolates from 85 BCs. In the remaining 17 BCs, obligate anaerobic species were isolated, for which AST is not routinely performed. All other isolates from monomicrobial and polymicrobial BCs were analyzed, with MDR bacteria being isolated in 30 of the 85 BCs (Table S1). Of the 25 isolated Enterobacterales (21 from monomicrobial and 4 from polymicrobial BCs), the most frequently isolated pathogens in our study, 15 showed a MDR phenotype. Most common among them was *E. coli*, of which 11 of 19 isolates showed MDR (Figure 2). Of the *E. coli* isolates, 3 were susceptible to all antimicrobial agents tested. The other Enterobacterales showing MDR were *K. pneumoniae* (*n* = 2/3), *Enterobacter cloacae* complex (*n* = 1/1), and *P. mirabilis* (*n* = 1/1). Of the *E. coli* isolates, 3 were phenotypically suspicious for ESBL production. Of the 23 coagulase-positive staphylococci (21 from monomicrobial and 2 from polymicrobial BCs), the second largest group of isolates, 6 (26.1%) showed a MDR phenotype. Three *S. pseudintermedius* isolates (13%) tested positive for methicillin resistance. Of the 17 *S. pseudintermedius* isolates*,* 2 were susceptible to all antimicrobials tested. All of the *S. aureus* and *Solanum coagulans* isolates tested negative for methicillin resistance and none were MDR. All 19 isolates of the third largest group, β-hemolytic streptococci (17 from monomicrobial and 2 from polymicrobial BCs), were susceptible to all β-lactams tested. Of the 19 isolates, 9 were susceptible to all antimicrobials tested (all *Streptococcus canis* isolates) and 2 (also *S. canis* isolates) were MDR. Most common among the β-hemolytic streptococci were fluoroquinolone (*n* = 7/19 [36.8%]) and tetracycline resistance (*n* = 7/19 [36.8%]). Different groups of pathogens had different MDR rates. The highest rates of MDR phenotypes were found in Enterobacterales (60.0%) and enterococci (*n* = 7/11 [63.6%]). Multidrug-resistant phenotypes were less frequently observed in coagulase-positive staphylococci (27.3%), followed by beta-hemolytic streptococci (10.5%). None of the 5 *Pasteurella* spp. isolates were MDR. The MDR pathogens were isolated in 61.1% (*n* = 22/36) of BCs taken from patients with antimicrobial administration in the 7 days before sampling in which AST was performed and in 12.8% (*n* = 6/47) of pathogens from BCs taken from patients with no antimicrobial administration in the 7 days before sampling. A significant association was found between prior antimicrobial treatment and the isolation of MDR bacteria (χ^2^(1) = 21.31; *P* < .001). The odds of a MDR bacterium being isolated were 10.74 times higher for dogs that had received antimicrobial treatment in the 7 days before sampling (95% CI, 3.62-31.86).

Acquired resistances against antimicrobial agents categorized as “D” by the EMA was found in 57 isolates (64.7%). Resistance against antimicrobial agents of this category was tested for in 88 isolates. The highest resistance rate was found in the Enterobacterales isolates (*n* = 21/25 [84.0%]). These were resistant to aminopenicillins (*n* = 21) and tetracyclines (*n* = 15). Resistance against antimicrobial agents from this category also was frequent in coagulase-positive staphylococci (*n* = 19/23, [82.6%]) and enterococci (*n* = 9/11, [81.8%]). Only 31 isolates were susceptible to all these antimicrobial agents (35.2%), including all 5 *Pasteurella* spp. isolates, both *Erysipelothrix* spp. isolates, and 12/19 of β-hemolytic streptococci (63.2%).

Acquired resistance to antimicrobial agents categorized as “B” and “C” was found in 24 (33.3% [72 tested]) and 40 (47.6% [84 tested]) of the isolates, respectively.

Susceptibility to antimicrobial agents categorized as “A” was tested in 11 isolates and no acquired resistance was detected.

### Concordance with cultures from other sampling sites

**Figure 3:**
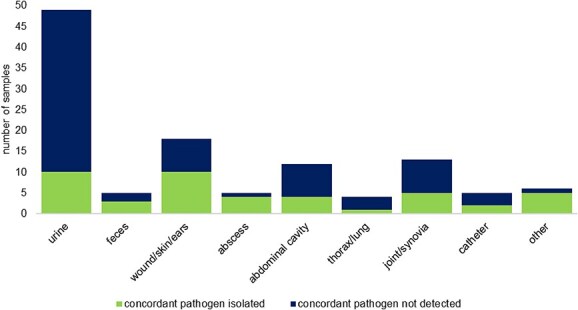
Number of cultures of other sampling sites during the same presentation episode from patients in which a blood culture was performed during the years 2014-2022 (Concordant pathogen: same pathogen identification and susceptibility testing results. In 2 urine samples with concordant pathogen identification results, antimicrobial susceptibility testing was not performed. These are included in the number of concordant pathogens).

In 65 (63.7%) of the 102 cases, in which clinically relevant pathogens were isolated from BCs, ≥ 1 additional culture from other sampling sites was performed during the same presentation episode. In 34 of these cases, 1 culture was performed from other samples, in 19 cases 2 cultures, in 7 cases 3 cultures, in 3 cases 4 cultures, and in 2 cases 5 cultures (Table S3). The most commonly sampled material was urine (Figure 3).

In 25 (38.5%) of the 65 cases, these additional cultures were negative, in 6 (9.2%) cases only discordant bacterial pathogens and in 32 (49.2%) cases concordant pathogens with the same AST patterns were detected, whereas in 2 (3.1%) cases isolates of the same species, but with different AST profiles, were found. In 11 of the 32 cases (34.4%) with concordant results, ≥ 1 additional discordant pathogen was isolated from the same culture. Obligate anaerobes were not isolated in any culture from other sampling sites. Coagulase-positive staphylococci were found in cultures from other sampling sites in 11 of the 16 cases in which these species were isolated from the BCs and ≥ 1 additional culture was performed (Table S2).

## Discussion

We analyzed BC results in dogs and elucidated the BC diagnostic process. The BC yield in our study (13.6%) is similar to reports in comparable veterinary studies.^4^^,^^16^^,^^26^ Older studies^27–29^ showed higher BC yields. Developments in BC processing in veterinary medicine render this comparison less compelling, and differences in the population being investigated and the frequency of performing BCs could explain this discrepancy. Limited data are available regarding BC contamination rates in veterinary medicine, although these are directly influenced by practitioners and have far-reaching consequences on patient care.^27^ A study on the effectiveness of multiple BCs in veterinary patients reported an overall contamination rate of 7.5%, while demonstrating that multiple BC sets increase contamination rates.^22^ This finding could be related to the larger number of BC bottles increasing the risk of one BC bottle being contaminated and a higher likelihood of pathogens with unclear pathogenicity being categorized as contaminants if only found in one BC bottle of a set. The performance standard in human medicine is set at ≤ 3%,^28^ putting the contamination rate found in our study in the acceptable range. Advancements in BC quality have led to suggestions of ≤ 1% as a limit.^8^ However, recent studies continue to report higher contamination rates from human medical clinics, ranging between 3.4% and 6.4%.^10^^,^^29^ We found polymicrobial growth in 7.8% of BCs, which is comparable to the only other recent report in dogs^26^ and to studies on BCs in humans.^6^^,^^30^ Older veterinary studies reported rates between 10% and 15%.^31–33^ Polymicrobial cultures pose a problem for clinicians, because antimicrobials must be aimed at the treatment of several pathogens,^34^ as well as for laboratories, because they can require longer or further subcultivation and therefore delay result reporting.^35^

### Sample processing

Only limited data on sample processing duration in veterinary medicine are available. A previous study found a median duration of 3 days from submission until final BC result reporting.^4^ Findings in human medicine are not directly applicable, because automated BC systems are commonly used to expedite processing, enabling constant surveillance of BC bottles by monitoring carbon dioxide production by microorganisms in the bottles.^36^ The CLSI standards for laboratories with automated BC systems recommend a preanalytical phase duration of ≤ 2 h.^28^ With the manual system utilized in the included cases, BC bottles were placed into an in-clinic incubator before transportation to the clinical microbiology laboratory. Thus, it may be more appropriate to apply this guideline to the time until initial placement of the BC bottle into the incubator, for which data were not available in our study. Nevertheless, identifying factors that may have led to a prolonged preanalytical phase is important, as such factors ultimately may contribute to delays in BC result reporting. Other influences on the duration of the preanalytical phase could be timing of sample submission and sample transport times. Recommendations for analytical phase duration of BC processing are not clearly stated for manual systems.^19^ Many approaches to expedite BC analytics, thereby decreasing the time until result reporting, have been explored.^37^^,^^38^ The day of the week of BC sampling had a significant impact on the duration of the preanalytical phase in our study. Studies in human medicine have shown lower BC yields and longer preanalytical phases for samples collected on weekends.^39^^,^^40^ Samples collected on weekends might wait longer until they are submitted for culture because of lower staff numbers. Because incubation starts in-clinic before submission, samples with longer preanalytical phases can have shortened analytical phases. Polymicrobial growth significantly increased the duration until final ID result reporting. As stated previously, polymicrobial growth can render the interpretation of results difficult and make further subcultivation steps necessary. Prior antimicrobial administration had no significant impact on sample processing duration. A recent study reported that prior antibiotic administration did not significantly affect BC yield, but the influence on sample processing duration was not explored.^12^ Studies have shown that initiation of empirical antimicrobial therapy significantly decreases the yield of cultures drawn shortly after treatment.^41^ However, 40.2% of the population in our study had a history of antimicrobial administration in the 7 days before sampling, and sample processing duration in this population was not significantly longer, leading to our recommendation not to forgo BC in patients with antimicrobial pretreatment. Sample processing duration was not significantly shorter for Gram-negative isolates. In a study in human medicine, the analytical phase of *E. coli*, a Gram-negative species, was shorter than for *S. aureus*, a Gram-positive species, likely because of the differences in their growth kinetics.^42^

In 81.4% of cases, the initial BC result was reported on day 1. All BC results were reported at this time to expedite result reporting and notify clinicians of a negative BC. If the BC remained negative thereafter, no further result was reported. Initial reporting that took longer than 1 day (18.6%) most likely was caused by sampling on a weekend or public holiday. Guidelines for human medicine state that de-escalation of antimicrobial treatment should be carried out as soon as possible.^43^ The high NPV of the first result in our study (0.974) showed that it is advisable to amend antimicrobial treatment based on this report. However, stopping antimicrobial treatment in patients in which a BC is reported positive after an initially negative result poses a risk and, therefore, this decision should be based on the clinician’s experience.

With the update of the Surviving Sepsis guidelines for human medicine in 2021, the previous recommendation of initiation of antimicrobial treatment within 1 h of presentation for patients with septic shock and suspected sepsis without shock was revised to 1 h for patients with septic shock and 3 h for patients with suspected sepsis without shock.^43^ This change should allow for the assessment of differential diagnoses and better antimicrobial stewardship.^43^^,^^44^ However, diagnosis of sepsis utilizing microbiological culture does not allow for such rapid result reporting, prompting research on alternative diagnostic methods, including molecular methods to detect sepsis pathogens,^45^ and the reliance on cytology results from other sampling sites to guide initial treatment.^46^ These approaches have their own limitations and currently are not suitable alternatives to BCs, rather acting as complementary methodologies.^47^

### Pathogen identification

The proportion of Gram-positive (67.7%) to Gram-negative (28.4%) isolates was comparable to the only other comparable veterinary study reporting this information, although only monomicrobial cultures were evaluated,^26^ and to older veterinary studies.^31–33^ Similar proportions have been reported in studies from human medicine.^48^^,^^49^

The most commonly isolated bacteria from monomicrobial BCs in our study (Enterobacterales, coagulase-positive staphylococci, β-hemolytic streptococci, and obligate anaerobic species) are species frequently isolated from BCs in dogs.^12^^,^^15^^,^^16^^,^^26^ In our study, *S. canis* was isolated in 17 BCs. Because other veterinary studies reported potential *S. canis* as β-hemolytic *Streptococcus* spp., it is not possible to determine whether we isolated this pathogen more frequently.^12^^,^^50^ Common isolates from BCs in humans frequently include coagulase-negative *Staphylococcus* (CoNS).^51^ Because only one BC bottle was collected per dog in our study, CoNS isolates were exclusively categorized as contaminants, because they are a part of the skin microbiota and the most common BC contaminant,^52^ potentially leading to their underrepresentation. Furthermore, no slow growing bacteria or yeasts were isolated, although these occur in BCs from humans.^51^ These pathogens not being reported in our study or other veterinary studies could mean that they play a minor role in veterinary medicine, or that the BC system used might not be suitable for detecting these organisms.

### Antimicrobial susceptibility testing

We applied the definition of MDR as defined previously.^21^ Increasing MDR rates of clinical Enterobacterales isolates, including the most commonly isolated species *E. coli*, make monitoring the resistance patterns of these species important. Despite this, no MDR rates in Enterobacterales isolated from BCs of dogs have been reported previously. The MDR rate of 60% in our study emphasizes the importance of AST to guide antimicrobial treatment. Of the Enterobacterales isolates, 3 were phenotypically suspicious for the production of ESBLs. This finding represents a slightly lower rate of ESBL-producing Enterobacterales isolates (1.6%) than the 3.6% reported in a previous study, which examined isolates from diseased companion animals in Europe.^53^ Methicillin-resistant staphylococci often display clinically relevant resistance to other antimicrobial agents, limiting treatment options.^54^ The rate of methicillin resistant *S. pseudintermedius* isolates in our study was 17.6%, compared with rates of 69.6% and 44.4% reported previously.^15^ This difference could be a result of epidemiological differences in the populations, because the previous study was conducted on isolates from companion animals outside of Europe. β-hemolytic streptococci isolated from companion animals commonly are susceptible to β-lactams.^55^^,^^56^ The *S. canis* isolates in our study were commonly resistant to tetracyclines (41.2%), a finding mirrored in other veterinary studies.^56^ Further resistance monitoring of this pathogen is vital, in part because of its zoonotic potential.^57^

Acquired resistance to antimicrobial agents in the EMA category “A” was tested in 11 of the isolates, none of which showed resistance. The only antimicrobial agent tested in this category was imipenem. Testing was performed in these low numbers and susceptibility was high because of the restricted use of these antimicrobial agents in animals and their full avoidance since June 2022, resulting in low to nonexisting direct selection pressure.

### Concordance with cultures from other sampling sites

No other veterinary study has included culture results from all other sites from the same presentation episode. Urine cultures often are taken when patients are presented with fever without a clear origin, and were, therefore, the most common culture from other sampling sites in our study. A previous study on the concordance of urine with BCs in dogs found poor to fair agreement,^14^ and another study found that positive urine cultures increased the odds of BC positivity (75% agreement).^12^ We did not find that urine cultures had better agreement with BCs compared to cultures from other sampling sites. However, the small number of cultures from each sampling site limited the ability to draw conclusions.

Concordant pathogens being isolated in 49.2% and discordant pathogens being isolated in 34.4% of the cases in which ≥ 1 other culture was performed indicated that pathogens isolated from different sampling sites during septic episodes cannot reliably be regarded as the causative agent, and thus, BC is recommended. This discordance could be a consequence of unrelated or incidental infections. These cultures cannot replace BCs but should be considered in the patient’s evaluation and treatment.

### Limitations

Because of the retrospective nature of our study, not all potentially relevant variables could be assessed, and a complete record was not available for all patients. Moreover, data regarding sample volume and sampling technique were not available. Because only positive cultures were included, influences on BC yield were not investigated. Sample size limited the potential for statistical analysis. Because of the selection of cases based on the available data, a priori power calculations were not performed. Possible population bias would need to be addressed in multicenter studies.

Pathogens were classified as contaminants to the best of our ability. However, accurate contaminant ID can be challenging,^58^ contaminant growth possibly could impact that of clinically relevant pathogens, and the collection of solitary BC bottles limited certainty. Collecting solitary BC bottles also can decrease BC yield, as shown in another veterinary study.^16^ Given the absence of a definitive gold standard for the calculation of the NPV, the reported results should be interpreted with caution, acknowledging the use of final BC results as a pseudo-gold standard.

Because only the dates of sample collection, receipt by the laboratory, and receipt of results were analyzed, the durations were reported in full days, limiting the accuracy. Because CLSI standards were updated during the 8 years included in the study, the comparability of the AST results may be slightly limited.

### Conclusion

The detection of MDR isolates and methicillin resistant staphylococci emphasizes the importance of BC diagnostic testing and rapid result reporting. Improving BC processing requires addressing BC yield, contamination rate, and BC management on weekends. Blood cultures should not be omitted in patients recently treated with antimicrobials, because they are more likely to yield MDR isolates and processing duration is unaffected. Culture results from other sampling sites may help guide antimicrobial treatment.

## Supplementary Material

aalaf057_Supplemental_Files

## Off-label antimicrobial declaration

The authors declare no off-label use of antimicrobials.

## Institutional animal care and use committee or other approval declaration

Ethical approval was not required for a retrospective study in accordance with the local legislation and institutional requirements. Written informed consent was not obtained from the owners for the participation of their animals in this study because it was not required (retrospective study). No dogs or clients are identifiable.

## Abbreviations

AST: antimicrobial susceptibility testing
BC: blood culture
BCs: blood cultures
BSIs: bloodstream infections
CLSI: Clinical and Laboratory Standards Institute
CoNS: coagulase-negative *Staphylococcus* spp.
EMA: European Medicines Agency
ESBL: extended-spectrum β-lactamase
ID: pathogen identification
MALDI-ToF MS: matrix assisted laser desorption/ionization time-of-flight mass spectrometry
MDR: multidrug-resistant
NPV: negative predictive value
