# Supplementary material for: Veterinary blood culture diagnostic testing: methodology and results from a German single-center study on dogs (2014-2022)
Source: J Vet Intern Med. 2026 Jan 21;40(1):aalaf057. doi: 10.1093/jvimsj/aalaf057 (PMC12881953; doi:10.1093/jvimsj/aalaf057)
Supplement: aalaf057_Supplemental_Files [file aalaf057_supplemental_files.zip › Supplemental_Table2_aalaf057.docx]

|  | Positive final result | Negative final result |
| --- | --- | --- |
| Positive initial result | 85 | N/A |
| Negative initial result | 17 | 648 |

Supplemental Table 2: Initial testing result and subsequent final result of positive canine BCs (n=102). False positives are marked with not applicable (N/A) as no initial positive result was amended to negative.
